# Efficacy of an herbal formulation LI10903F containing *Dolichos biflorus* and *Piper betle* extracts on weight management

**DOI:** 10.1186/1476-511X-11-176

**Published:** 2012-12-27

**Authors:** Krishanu Sengupta, Atmatrana T Mishra, Manikeshwar K Rao, Kadainti VS Sarma, Alluri V Krishnaraju, Golakoti Trimurtulu

**Affiliations:** 1 Laila Impex R&D Center, Unit-I, Phase-III, J. Autonagar, Vijayawada, India; 2 Department of Internal Medicine, ASR Academy of Medical Sciences, Eluru, India; 3 Suraksha Health Village, Gurunanak Nagar, Vijayawada, India; 4 Department of Statistics, SV University, Tirupati, India

**Keywords:** Adiponectin, *Dolichos biflorus*, Ghrelin, Human clinical study, LOWAT, *Piper betle*, Weight loss

## Abstract

**Background:**

A novel herbal formulation LI10903F, alternatively known as LOWAT was developed based on its ability to inhibit adipogenesis and lipogenesis in 3T3-L1 adipocytes model. The clinical efficacy and tolerability of LI10903F were evaluated in an eight-week, randomized, double-blind, placebo-controlled, clinical trial in 50 human subjects with body mass index (BMI) between 30 and 40 kg/m^2^ (clinical trial registration number: ISRCTN37381706). Participants were randomly assigned to either a placebo or LI10903F group. Subjects in the LI10903F group received 300 mg of herbal formulation thrice daily, while subjects in the placebo group received 300 mg of placebo capsules thrice daily. All subjects were provided a standard diet (2,000 kcal daily) and participated in a moderate exercise of 30 min walk for five days a week. Additionally, the safety of this herbal formulation was evaluated by a series of acute, sub-acute toxicity and genotoxicity studies in animals and cellular models.

**Results:**

After eight weeks of supplementation, statistically significant net reductions in body weight (2.49 kg; p=0.00005) and BMI (0.96 kg/m^2^; p=0.00004) were observed in the LI10903F group versus placebo group. Additionally, significant increase in serum adiponectin concentration (p=0.0076) and significant decrease in serum ghrelin concentration (p=0.0066) were found in LI10903F group compared to placebo group. Adverse events were mild and were equally distributed between the two groups. Interestingly, LI10903F showed broad spectrum safety in a series of acute, sub-acute toxicity and genotoxicity studies.

**Conclusions:**

Results from the current research suggest that LI10903F or LOWAT is well-tolerated, safe and effective for weight management.

## Introduction

Studies indicate that obesity-related risk factors are preventable and even ameliorable through weight loss and long-term weight management programs [1-3]. There are three major weight management strategies: 1) life style intervention such as diet and exercise as well as dietary supplements, 2) pharmacotherapy, and 3) bariatric surgery for morbidly obese subjects. While pharmacotherapy may be effective, it also has significant adverse effects and limitations [4]. Since obesity is associated with an imbalance between food intake and energy expenditure, a long-term lifestyle change including healthy diet and exercise in conjunction with effective weight management supplements may present a cost-effective intervention for obesity [5-8]. In fact, informed consumers are increasingly seeking dietary supplements which have been shown to be efficacious and safe in promoting weight loss [9].

In order to develop an effective and safe weight loss ingredient, we screened extracts of more than four hundred medicinal plants for their ability to inhibit adipogenesis in 3T3-L1 mouse adipocytes. Among the candidate plant extracts tested, *Piper betle* leaf extract and *Dolichos biflorus* seed extract showed potent anti-adipogenic efficacy. These two extracts were selected for further studies by combining individual extracts at different ratios. A combination of *P. betle* leaf extract and *D. biflorus* seed extract in a ratio of 2:3, LI10903F also known as LOWAT demonstrated greater anti-adipogenic as well as lipolytic activities compared to the individual extracts.

Here we report the *in vitro* development of LI10903F or LOWAT and the results of its safety studies. In addition, we present the findings of a randomized, double-blind, placebo-controlled clinical study demonstrating the tolerability and short term efficacy of LI10903F on body weight management.

## Materials and methods

### Plant materials

#### *Piper Betle*

The dried plant material (1kg) of *Piper Betle* leaves was pulverized to course powder and extracted with 60% alcohol at 70°C for 2h. The extraction process was repeated with the residue for three times. The extracts were combined, filtered and evaporated under reduced pressure at 50-60°C to obtain a residue (120g).

#### *Dolichos biflorus*

The dried seeds (1kg) of *Dolichos biflorus* were pulverized to course powder and extracted with 90% alcohol for 1h. The extract was filtered and the residue was subjected for repeated extraction for 3 times. The extracts were combined and evaporated under vacuum to obtain a residue (100g). LI10903F is a 2:3 combination of *P. betle* leaf aqueous-alcohol extract and *D. biflorus* seed alcohol extract.

### Chemicals and reagents

Isopropyl alcohol, insulin, dexamethasone, 3-isobutyl-1-methylxanthine (IBMX), Oil red O, Dulbecco’s modified Eagle’s medium (DMEM), glutamine, glucose, penicillin, streptomycin were obtained from Sigma Chemical Co. (St. Louis, MO). Fetal bovine serum (FBS) was purchased from Hyclone (Logan, UT). All other reagents were purchased from Sigma Chemical Co. unless otherwise indicated.

### In vitro studies

#### Cell culture and treatment

Mouse pre-adipocyte fibroblasts 3T3-L1 cells were obtained from American Type Culture Collection (Manassas, VA) and cultivated in maintenance medium comprised of DMEM supplemented with 10% FBS, 100 U/ml penicillin, 100 μg/ml streptomycin, 1 mM sodium pyruvate and 4.5 g/L D-glucose.

#### Adipogenesis assay

Equal number of 3T3-L1 cells (60,000 cells per well) was seeded in each well of 24-well culture plates. Cells were pre-treated with different concentrations of herbal extracts or formulations for 2h and incubated further with the differentiation medium containing 500 nM insulin, 1 μM dexamethasone, and 0.5 mM IBMX for 48h. Thereafter, cells were maintained in the post-differentiation medium (DMEM containing 100nM insulin) in presence or absence of LI10903F for further 8 days. The control cultures received only 0.1% (v/v) DMSO as the vehicle. The intracellular lipid accumulation was measured by staining the cells with Oil Red O stain following the method described earlier [10].

#### Lipolysis assay

The intracellular lipid breakdown efficacy of herbal extracts and their formulations was evaluated by measuring the released glycerol in the 3T3-L1 culture supernatants. Equal number of mature adipocytes was treated with different concentrations of either *P. Betle* or *D. biflorus* extracts or their formulations in phenol red-free DMEM supplemented with 2% bovine serum albumin (BSA) for 4h. Released glycerol in the cell free culture supernatants was measured using the Adipolysis Assay Kit (Millipore, Billerica, MA) as described previously [10].

#### Toxicity studies

Acute oral and 28-day dose-dependent oral toxicity studies were conducted at Laila Impex R&D Centre (Vijayawada, India). *In vitro* chromosome aberration test and mammalian erythrocyte micronucleus test were conducted at Shriram Institute for Industrial Research (Delhi, India). Ames bacterial reverse mutation assay was conducted at Vimta Labs Limited (Hyderabad, India). All studies complied with the Good Laboratory Practice (GLP) Standards, and were done according to the OECD guidelines for the testing of chemicals. The details of these procedures were described previously by Sreejayan et al. [11].

#### Acute oral toxicity

An acute oral toxicity study was conducted following the protocol as described earlier [11]. Three Sprague–Dawley (SD) female rats, nulliparous and non-pregnant (age: 12 weeks; 205–230 g body weight at study initiation) were administered orally with a single dose of 5000 mg/kg of LI10903F. All the animals were observed for mortality, signs of gross toxicity, and behavioral changes on the day of dosing and thereafter for 14 days. All animals were euthanized using anesthetic ether at the end of the observation period and subjected to necropsy.

#### 28-day sub-chronic toxicity

A twenty-eight day sub chronic toxicity study was conducted following the protocol as described earlier [12]. Forty healthy Sprague Dawley rats (20 males and 20 females) were equally divided into four groups. Each group of animals (n=10) was supplemented with 50 or 250 or 2500 mg/kg LI10903F or vehicle (1% CMC). The animals were given LI10903F for 28 consecutive days by oral gavage using a ball-tipped gavage needle attached to an appropriate syringe. Animals were monitored for mortality, toxicity, and behavioral changes throughout the study period. Body weights were recorded before dosing (Day 0), and at weekly intervals thereafter.

#### Ames test: reverse mutation assay

The reverse mutation test measures the ability of LI10903F to induce reverse mutation at selected histidine loci in five tester strains of *Salmonella typhimurium* TA 98, TA 100, TA 102, TA 1535, and TA 1537 (Molecular Toxicology, Boone, NC). In the dose-range finding study, the herbal blend was tested at the dose levels of 39.06, 78.13, 156.25, 312.50, 625, 1250, 2500, and 5000μg/mL in presence (10% S9 fraction v/v) or absence of metabolic activation system.

#### Mammalian erythrocyte micronucleus test

A total of 60 healthy Swiss albino mice (30 males, 30 females) of approximately 8–12 weeks age were randomly assigned to three main study groups (n=20) with equal number of either gender of animals in each group. Animals in Group 1 were administered orally with 2000 mg/kg of LI10903F; animals in Group 2 and Group 3 were administered with corn oil (negative control) and 40 mg/kg cyclophosphamide via i.p., respectively. Animals were observed for toxicity or mortality. Five males and five females from each group were sacrificed after 24h, and similarly five males and five females after 48h of dose administration. The bone marrow smears were prepared from femur, stained with May and Graunwald stain and examined for polychromatic erythrocytes (PCE) under 40X objective of a Nikon Eclipse TS 100 microscope (Nikon Corporation, Japan).

#### Mammalian chromosome aberration test

Whole blood was collected in heparinized vial from two healthy male human volunteers. The test was conducted on isolated lymphocytes at 1000, 2500 and 5000 μg/mL of LI10903F with or without metabolic activation. Cultures treated with mitomycin-C or cyclophosphamide served as positive control, whereas, culture treated with 0.1% DMSO as negative control. Colchicine was added to the cultures 2h prior to harvesting. After 18h of treatment with LI10903F, lymphocytes were harvested for assessing chromosomal aberration. Metaphase preparations were stained with Giemsa and aberrations classified and scored under 40X objective of a Nikon Eclipse TS 100 microscope (Nikon Corporation, Japan).

#### Clinical study material

The study material LI10903F or LOWAT is a blend of 60% alcohol extract of *P. bettle* leaves standardized to 6% polyphenols plus 90% alcohol extract of *D. biflorus* seeds standardized to 4% γ-glutamyl phenylalanine mixed in 2:3 ratio. For clinical study, three hundred milligrams of LI10903F was encapsulated in opaque capsules with 200 mg of exicipient. A combination of micro crystalline cellulose and Magnesium stearate (<2%) was used as the excipient. Placebo capsules contained only exicipient, and were identical in appearance, size, weight and color to the active capsules. The formulation was provided by InterHealth Nutraceuticals (Benicia, CA).

#### Trial site and recruitment of subjects

This randomized, double-blind, placebo controlled, clinical trial (RCT) was performed at Alluri Sitarama Raju Academy of Medical Sciences (ASRAM), Eluru, Andhra Pradesh, India (clinical trial registration number: ISRCTN37381706). The protocol was evaluated and approved by the ASRAM Institutional Review Board (IRB). IRB approval Reference number, 07-001/IB/PHF OB.

One hundred forty five subjects participated in a questionnaire-based screening process. Inclusion criteria were: adults (21–50 years), BMI ≥ 30 kg/m^2^, female participants should not be pregnant, willingness to follow method of birth control if premenopausal. Exclusion criteria were: morbidly obese (BMI > 40 kg/m^2^), history of thyroid disease, cardiovascular disease, diabetes, hepatitis, pancreatitis, lactic acidosis or hepatomegaly with steatosis, motor weakness, peripheral sensory neuropathy; allergies to spices and herbal products; using weight loss medications, laxatives or diuretics taken solely for the purpose of weight loss; recent unexplained weight loss or gain; HIV positive; any evidence of organ dysfunction or any clinically significant deviation from normal that could impact subjects well-being or clinical outcome. Of 145 candidates screened 50 subjects who met eligibility criteria were enrolled in the study. Each participant voluntarily signed the IRB-approved, informed-consent form. At baseline visit, the subjects were randomized into either the placebo or LI10903F group.

#### Study design

At baseline visit, all subjects were provided with LI10903F or placebo capsules, compliance cards, and dates for follow-up evaluations. A standard diet (2,000 kcal daily) was provided (breakfast, lunch and dinner) deriving 61% kcal from carbohydrate, 14% from protein and 25% from fat. The subjects were required to walk 30 min, five days a week under the supervision of a physical trainer. The subjects were instructed to take 3 capsules a day, one each 30 min before breakfast, lunch and dinner. All subjects completed a medical history questionnaire at baseline and compliance reports during follow-up evaluations at 14, 28 and 56 days. Subjects were assessed for body weight, height, waist plus hip circumference and vital signs at baseline and subsequent follow-up visits. At all study visits, blood samples were collected for hematological and biochemical evaluations, and urine samples for urinalysis. Reduction in body weight and BMI were considered as the primary study outcome parameters of the study. Sample size calculations were done using power analysis based on previous obesity study report [13]. We estimated that more than 95% power at the two-tailed α level of 0.05 would be provided to test the significance of weight reduction over placebo with 25 subjects per group.

#### Serum adipokine assay

Serum adiponectin and ghrelin levels were determined using the human Adiponectin ELISA kit (Millipore, St. Charles, MO) and the human Ghrelin ELISA kit (Millipore, St. Charles, MO). The detection sensitivity of adiponectin and ghrelin assays was 0.78 ng/ml and 8 pg/ml, respectively.

#### Statistical analysis

Mean values were compared with analysis of variance (ANOVA) on variables for between-group differences at each individual time point with repeated measurements using group and time as main effects and baseline of the variables and gender as covariate. For baseline (week 0) comparisons, a *t*-test was used. For inter group comparisons, a p-value < 0.05 is considered to be statistically significant. All comparisons reported are between the placebo and LI10903F groups at each specified time point. All analyses were performed with SYSTAT (Windows version 11.0; San Jose, CA).

## Results

### LI10903F inhibits adipogenesis in 3T3-L1 adipocytes

**Figure 1:**
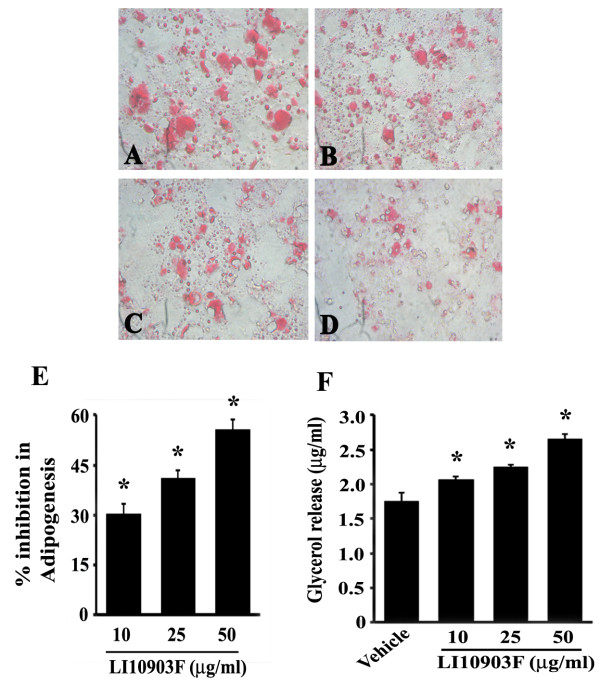
**3T3-L1 cells were induced to differentiate in presence or absence of different concentrations of LI10903F.** Intracellular Lipids were stained with Oil Red O. Representative photomicrographs show lipid accumulation in the cells treated with 0.1% DMSO as vehicle control (**A**); cells treated with 10, 25 and 50μg/ml LI10903F (**B**, **C**, and **D**, respectively). **Panel E,** bar diagram represents percent inhibition of adipogenesis (mean ± SD) in LI10903F treated 3T3-L1 cells relative to the vehicle control cultures (n=3). Bound Oil Red O dye was eluted from treated adipocytes with 100% isopropyl alcohol, and the OD was read at 550 nm. * Denotes significantly different compared to control cultures, p<0.05. **Panel F,** 3T3-L1 adipocytes were treated with either different concentrations of the herbal blend (10, 25 or 50μg/mL) or 0.1% DMSO (control). Lipid breakdown was estimated based on the amount of glycerol released in the culture medium. Bar diagram represents released glycerol (mean ± SD) in LI10903F treated and vehicle treated cultures (n=3). *Denotes significantly different compared to vehicle treated controls, p<0.05.

A panel of Indian herbs indicated for various medicinal uses in Ayurveda was screened for anti-adipogenesis activity following a standard screening protocol [14]. Based on the ability to inhibit lipid accumulation in 3T3-L1 adipocytes, we selected 60% alcohol extract of *P. Betle* leaf and 90% alcohol extract of *D. biflorus* seeds. Table 1 shows percent inhibition of adipogenesis by these extracts at 10, 25 and 50 μg/ml. Thereafter, with an intension to develop a synergistic formulation with better anti-adipogenic properties than the stand alone, these two extracts were combined at different ratios such as 1:1, 2:1, 1:2, 2:3, 3:2, 1:4, 4:1. Interestingly, we observed that the formulation composed of 2 parts of *P. Betle* leaf extract and 3 parts of *D. biflorus* seeds extract demonstrated significantly better anti-adipogenic activity than the individual extracts (Table 1). Lipid staining in 3T3-L1 adipocytes revealed that LI10903F inhibited adipocyte differentiation and lipid accumulation in a dose-dependent manner (Figure 1, A-D). In comparison with the vehicle (0.1% DMSO) treated cultures, LI10903F treatment exhibited 30.65% (p=0.0079), 40.95% (p=0.0021) and 55.80% (p=0.0001) inhibition in adipogenesis at 10, 25, 50 μg/mL, respectively (Figure 1E).

**Table 1:** Herbal extracts inhibit adipogenesis in 3T3-L1 cells

**Treatments**	**Treatment conc.**	**Percent of inhibition in Adipogenesis**				**SD**	P values*	
		**1**^**st**^**Assay**	**2**^**nd**^**Assay**	**3**^**rd**^**Assay**	**Average**		**LI10903F vs.*P. betel***	**LI10903F vs.*D. biflorus***
*P. betel*	10μg/ml	23.79	20.80	21.28	21.96	2.29		
	25μg/ml	27.50	24.85	26.06	26.13	1.34		
	50μg/ml	42.98	42.78	41.01	42.25	3.24		
*D. biflorus*	10μg/ml	26.68	25.98	25.38	26.01	1.68		
	25μg/ml	26.56	33.87	30.07	30.16	3.66		
	50μg/ml	37.50	40.99	38.97	39.15	1.80		
LI10903F	10μg/ml	27.91	33.76	30.29	30.65	3.07	0.0108	0.0562
	25μg/ml	38.52	43.52	40.82	40.95	2.52	0.0008	0.0134
	50μg/ml	52.89	58.96	55.54	55.80	3.11	0.0019	0.0012

* Paired t-test, P<0.05 Significant.

### LI10903F promotes lipolysis in 3T3-L1 adipocytes

The effect of LI10903F on lipolysis (measured as glycerol secretion) in matured 3T3-L1 adipocytes showed a dose-dependent increase compared to the 0.1% DMSO treated control cultures (Figure 1F). Adipocytes treated with 10, 25 and 50μg/mL LI10903F exhibited 18.10% (p=0.0123), 28.26% (p=0.0023) and 51.39% (p=0.0003) increase in glycerol content in culture medium, in comparison with the vehicle treated cultures. This observation indicates that LI10903F increases lipolysis or fat mobilization from the adipocytes.

### Systemic toxicity studies demonstrate safety of LI10903F

In acute oral toxicity study, administration of the herbal blend at a limit dose of 5000 mg/kg in female SD rats did not show any significant abnormalities in all examined tissues. Based on these results, the oral LD_50_ of the herbal blend was concluded to be > 5000 mg/kg body weight (Table 2).

**Table 2:** Result summary of toxicity evaluation studies on LI10903F

**Test**	**Result**
Acute oral toxicity	LD_50_ > 5,000 mg/kg
28-day sub-acute toxicity	NOAEL > 2,500 mg/kg
Ames bacterial assay	Non-mutagenic
Mouse micronucleus test	Non-mutagenic
Chromosome aberration test	Non-mutagenic

In 28-day repeat dose toxicity study, oral administration of LI10903F to male and female SD rats at a dose of 0, 50, 250 or 2500 mg/kg for 28 days did not show any toxicologically significant effects. There were no significant adverse effects observed in serum biochemistry, hematology or microscopic tissue examination (data not shown). Based on these data, the no-observed-adverse effect-level (NOAEL) for the herbal blend was considered to be at least 2500 mg/kg body weight in male and female SD rats (Table 2).

### LI10903F is non-mutagenic and non-genotoxic

In reverse mutation assay, five strains of *S. typhimurium* were used to evaluate the mutagenic potential of the LI10903F in the presence and absence of metabolic activation. In all doses including the highest dose of 5000 μg/ml, the mutant frequency was below 1.5. According to the OECD guidelines, these observations indicate that LI110903 is non-mutagenic. This herbal formulation did not affect mitotic index at up to the dose of 5000 μg/ml, and also failed to induce structural chromosomal damage in the immature erythrocytes. In addition, micronucleus test conducted in mammalian erythrocytes demonstrates that LI10903F is not cytotoxic at the highest dose level at 2000 mg/kg. Table 3 depicts a summary of the cytogenetic effects of LI10903F at the highest dose levels tested in Erythrocyte micronucleus test and chromosomal aberration test, respectively. In summary, these observations together suggest that LI10903F is safe for consumption and also non mutagenic and non-genotoxic. The overall results obtained from various toxicity studies are summarized in Table 2.

**Table 3:** Summary of cytogenetic effect of LI10903F in mammalian cells

**Test**	**Test Parameters**	**Negative control**	**LI10903F 2000mg/kg**	**Cyclophosphamide 40 mg/kg**
**Mammalian Erythrocyte Micronucleus Test**	No. of immature Erythrocytes scored	2000	2000	2000
	No. of Micronuclei present in immature erythrocytes	0.60±0.54	0.80±0.49	45.80±4.44
**Test**	**Test Parameters**	**Negative control**	**LI10903F 5000μg/ml**	**Mitomycin C 0.2 μg/ml**
**Mammalian Chromosome Aberration Test**	No. of lymphocytes scored	100	100	100
	% Numerical Aberrations	0	1.50±0.71	30.50*±7.78
	No. of cells with one structural aberration	0	1.50±0.71	39.00*±5.66
	No. of cells with two structural aberrations	0.50±0.71	0.50±0.71	38.00*±4.24
	% Mitotic Index	3.65±0.12	2.65±0.21	1.30*±0.14

ANOVA, * Significant (p<0.05).

### LI10903F reduces body weight and BMI in obese subjects

Baseline demographic characteristics of subjects with respect to gender, age, weight, BMI and height were similar between the placebo and LI10903F supplemented groups (Table 4). In comparison with the baseline, LI10903F group experienced 3.11±0.23 kg and 4.28±0.40 kg reductions of body weight at 4 and 8 weeks, respectively. Whereas, 1.58±0.22 kg and 1.79±0.35 kg body weight were reduced in placebo group at 4 and 8 weeks, respectively (Table 5). The net reductions in body weight in LI10903F group were 1.53 kg and 2.49 kg at 4 and 8 weeks, respectively (Table 5). Similarly, significant decrease in BMI (1.19±0.08 and 1.66±0.15 kg/m^2^ at 4 and 8 weeks, respectively) was observed in LI10903F group compared to the baseline (Table 5). Placebo group experienced BMI reductions of 0.63±0.09 and 0.70±0.13 kg/m^2^ at 4 and 8 weeks, respectively. The significant net reductions in BMI in LI10903F group were 0.56 and 0.96 kg/m^2^ at 4 and 8 weeks, respectively (Table 5).

**Table 4:** Demographic and baseline characteristics

**Characteristics**	**Placebo (n = 20)**	**LI10903F (n=20)**	***P*value**
Number of females (%)	15 (75)	12 (60)	NA
Age (years)	37.15 ± 1.51	39.25 ± 1.35	0.307
Height (meters)	1.60 ± 0.02	1.60 ± 0.02	0.993
Body weight (kg)	84.97 ± 2.80	86.83 ± 2.95	0.650
Body mass index (kg/m^2^)	33.01 ± 0.73	33.67 ± 0.80	0.546

Values are expressed as mean ± SEM.

**Table 5:** Reduction of body weight and body mass index (BMI) at 2, 4, and 8 weeks

**Parameters**	**Week**	**Placebo (n=20)**	**LI10903F (n=20)**	**Net reduction**^**a**^	***P*-value**
Reduction in body weight (kg)	2	0.79 ± 0.23	1.41 ± 0.25	0.62	0.10859
	4	1.58 ± 0.22	3.11 ± 0.23	1.53	0.00002*
	8	1.79 ± 0.35	4.28 ± 0.40	2.49	0.00005*
Reduction in BMI (kg/m^2^)	2	0.31 ± 0.08	0.53 ± 0.09	0.22	0.11209
	4	0.63 ± 0.09	1.19 ± 0.08	0.56	0.00003*
	8	0.70 ± 0.13	1.66 ± 0.15	0.96	0.00004*

Values represents mean ± SEM. *Significant difference between supplement and placebo groups.

^**a**^Net reduction = Herbal minus placebo.

### LI10903F Modulates serum adiponectin and ghrelin levels

LI10903F supplementation for 8 weeks resulted in 15.35% (p=0.0076) higher serum adiponectin concentration as compared to placebo (Table 6). This finding suggests that the herbal formula LI10903F may influence reducing the fat store in obese individuals in agreement with the earlier observation that serum adiponectin is inversely correlated to percent of body fat [15]. Interestingly, LI10903F supplementation also resulted in 20.85% (p=0.0066) reduction of serum ghrelin levels than in placebo after 8 weeks. Ghrelin is a peptide hormone predominantly produced in stomach and is known to potently enhance feeding and weight gain [16]. Its reduction in circulating levels may curb appetite thereby promoting weight loss.

**Table 6:** Serum levels of adiponectin and ghrelin after 8 weeks of supplementation

**Parameter (Units)**	**Placebo (n = 17)**	**LI10903F (n = 17)**	**Percent Difference Relative to Placebo†**	***P-*value (Between Groups)**
Adiponectin (μg/ml)	6.71 ± 0.23	7.74 ± 0.30	15.35	0.0076*
Ghrelin (pg/ml)	52.10 ± 3.22	43.11 ± 0.76	−20.85	0.0066*

Results are presented as Mean ± SEM. *Significant difference between placebo and LI10903F groups.

†Percent Difference Relative to Placebo = (LI10903F group - Placebo) / Placebo x 100.

### Safety assessment

Analyses of various hematologic parameters plus other markers associated with liver, heart, kidney and metabolic function showed no significant difference between the placebo and the LI10903F groups (Table 7). No changes were observed for multiple vital signs that included systolic and diastolic blood pressure and pulse rate (Table 7). Triglyceride levels were significantly (p < 0.05) reduced by herbal supplementation as compared to the placebo (Table 7). This is consistent with improved lipid profile after weight loss [17].

**Table 7:** Changes in biochemical parameters after 8 weeks of supplementation compared to baseline

**Parameter (Units)**	**Placebo (n = 20)**	**LI10903F (n = 20)**	***P*value**
**Liver Function**			
SGOT (U/L)	−1.50 ± 3.63	−2.55 ± 6.36	0.397
SGPT (U/L)	−7.65 ± 2.65	−11.70 ± 4.65	0.839
**Cardiac Function**			
Systolic BP (mm Hg)	−2.40 ± 3.00	−12.53 ± 3.92	0.105
Diastolic BP (mm Hg)	−0.70 ± 2.07	−5.05 ± 2.89	0.975
Pulse rate (beats/min)	0.80 ± 1.48	−0.11 ± 1.06	0.786
**Metabolic panel**			
Cholesterol (mg/dL)	12.20 ± 7.47	−1.2 ± 6.83	0.119
Triglycerides (mg/dL)	−8.76 ± 17.32	−26.50 ± 30.27	0.041*
LDL (mg/dL)	−23.43 ± 7.08	−11.64 ± 13.37	0.155
HDL (mg/dL)	−3.04 ± 1.23	1.33 ± 4.44	0.873
Fasting Glucose (mg/dL)	−6.20 ± 10.68	−16.15 ± 7.35	0.445

Results are presented as Mean ± SEM. *Statistical significant difference is between the placebo and the herbal groups.

Abbreviations: SGOT=Serum glutamic oxaloacetic transaminase; SGPT=Serum glutamic pyruvic transaminase; LDL=Low density lipoproteins; HDL=High density lipoproteins.

Some minor adverse events were noted during the course of the 8-week study period, which include gastric irritation in two subjects (one from each group), abdominal pain in one placebo subject, back pain in one subject belongs to LI10903F group, and burning sensation at rectum in one placebo subject. The subjects who reported these minor events were distributed evenly throughout the placebo and active groups.

### Dropouts

Ten subjects, five from each group, dropped from the study and were lost to follow-up. None of the drop-outs were associated to an adverse event. As pre-defined in the study protocol, drop-outs were excluded from all statistical analyses.

## Discussion

To develop an efficacious and safe herbal formulation that promotes healthy weight loss, we screened a series of extracts derived from ayurvedic medicinal plants for their anti-adipogenic properties. We found that extracts from *P. bettle* leaves and *D. biflorus* seeds blended in a ratio of 2:3, and exhibited potent *in vitro* anti-adipogenic and lipolysis promoting activities. We then conducted a series of *in vivo* and *in vitro* safety studies that demonstrated this herbal formulation LI10903F or LOWAT is safe, non-mutagenic and non-genotoxic with an acute oral LD_50_ of > 5,000 mg/kg and NOAEL > 2,500 mg/kg body weight. Interestingly, LI10903F also passed through other safety studies conducted for acute dermal toxicity, primary dermal irritation, and primary eye irritation in various animal models (unpublished observation). These findings encouraged and led us to conclude that the herbal formulation was safe, and can be evaluated for its weight management efficacy in human clinical trials.

The efficacy and tolerability of this herbal blend was evaluated in a randomized double blind placebo controlled clinical study in overweight and obese subjects (clinical trial registration number: ISRCTN37381706). Our observations from this study are that the herbal blend, when supplemented together with a standard diet and moderate daily exercise, promotes a significantly higher reduction of body weight and BMI than food management and exercise alone. In the clinical study, subjects received a 900mg daily dose of either LI10903F or placebo for 8 weeks. At the end of the study, the herbal formulation supplemented subjects experienced a greater reduction in body weight (2.4-fold) and BMI (2.37-fold) than placebo. This higher net loss of body weight can be attributed to the herbal blend supplementation rather than to dietary alteration or moderate exercise. This is because all the subjects followed a standard calorie diet of 2,000 kcal per day as opposed to being placed on a hypocaloric diet [18-20]. By taking this approach, we wanted to mitigate the failure of individuals to comply with their dietary restrictions [21]. Therefore, we believe that the results derived from this study are generally applicable to situations in which individuals adapt a more healthy living style that includes moderate exercise and diet control.

We also observed a significant reduction in body weight and BMI as early as four weeks in herbal supplemented group. A significantly higher 3.0-fold reduction of triglycerides was also observed in the herbal group versus the placebo at 8 weeks. In addition, it is interesting to observe that the other lipid parameters such as total cholesterol, LDL and HDL were noticeably improved in the herbal formulation supplemented group in comparison with the placebo. These improvements are not statistically significant. We have noticed high standard error values in placebo and treatment groups; this might be a possible reason for failure to achieve the statistically better improvements in such parameters. We believe that further clinical study with larger group sizes can definitely address this issue. However, this improvement in blood lipid profile may be a secondary beneficial effect of weight loss [19].

Previous studies report that adiponectin, an adipokine hormone predominantly secreted by white adipose tissue (WAT) plays an important role in pathogenesis of obesity and type II diabetes [22]. Circulating adiponectin levels are lower in subjects with obesity, diabetes and cardiovascular diseases than those of healthy control subjects [23]. At the end of the present study, we found that the serum adiponectin levels were significantly higher in subjects supplemented with LI10903F than with placebo. This result provides a mechanistic basis for the effects of the herbal formulation on body fat metabolism. Interestingly, at the end of the study we also observed that the serum levels of ghrelin was significantly lower in LI10903F supplemented group than in placebo. Ghrelin is predominantly produced by the stomach in humans [24], it promotes glucose oxidation and lipogenesis [25]. Ghrelin is a key hormone involved in controlling hunger [26] by exerting its appetite stimulating effects [27]. Higher level of circulating ghrelin level is correlated with increased food intake and adiposity in both animals and humans [25,28]. In agreement with the previous findings our observation suggests that LI10903F or LOWAT might exert its weight reducing effects via regulation of food intake. In addition, our *in vitro* experiments also suggest that LI10903F might reduce body fat by inhibiting the differentiation process from pre-adipocytes to mature adipocytes and enhancing fat mobilization from the mature adipocytes.

Overall, we did not observe any major adverse events from the use of the herbal blend. Only a few minor adverse events were reported and they were evenly distributed between the placebo and the herbal groups. There was no change in blood markers related to liver, kidney and heart function, nor did we note any changes in subject vital signs.

In conclusion, supplementation with the herbal blend at 900 mg per day resulted in statistically significant reductions in body weight and BMI that exceeded those achieved via diet management and moderate exercise alone. Its significant effect on body weight reduction was seen starting at week 4. Further, serum adiponectin levels were significantly higher in herbal group than in placebo, whereas, serum ghrelin levels were significantly lower in herbal group than in placebo. Analyses of various safety parameters indicate that the herbal blend appears to be well-tolerated for human consumption. We conclude that this herbal blend appears to be an effective ingredient for weight management.

## Competing interests

This study is funded by Laila Impex R&D Center, India. KS, TG and KVA are employees of Laila Impex R&D Centre, Vijayawada, India. AM is an employee of ASRAM, Eluru, India. KM is an Ayurvedic Physician at Suraksha health village, Vijayawada, India. KVSS is a Professor in Department of Statistics, SV University, Tirupati, India.

## Authors’ contribution

KS contributed to the design of the project and data analysis, and was primarily responsible for writing the manuscript. AVK contributed to the design of the project and data analysis, subject recruitment and management, data collection and writing of manuscript. ATM worked with subjects to obtain informed consent, conducted clinical evaluations, took samples and evaluated therapeutic responses. TG contributed in the development of the formulation and coordinated the study. MKR associated with study as a consultant Ayurvedic Physician. KVSS is a consultant statistician and contributed in clinical data analysis. All authors read and approved the final manuscript.
